# *In vitro* and *in vivo* Evaluation of *in silico* Predicted Pneumococcal UDPG:PP Inhibitors

**DOI:** 10.3389/fmicb.2020.01596

**Published:** 2020-07-15

**Authors:** Freya Cools, Dhoha Triki, Nele Geerts, Peter Delputte, Denis Fourches, Paul Cos

**Affiliations:** ^1^Department of Pharmaceutical Sciences, Laboratory for Microbiology, Parasitology and Hygiene (LMPH), University of Antwerp, Antwerp, Belgium; ^2^Department of Chemistry, Bioinformatics Research Center, North Carolina State University, Raleigh, NC, United States

**Keywords:** *Streptococcus pneumoniae*, GalU, *in silico* modeling, virulence, *Galleria mellonella*, novel drug target

## Abstract

Pneumonia, of which *Streptococcus pneumoniae* is the most common causative agent, is considered one of the three top leading causes of death worldwide. As seen in other bacterial species, antimicrobial resistance is on the rise for this pathogen. Therefore, there is a pressing need for novel antimicrobial strategies to combat these infections. Recently, uridine diphosphate glucose pyrophosphorylase (UDPG:PP) has been put forward as a potential drug target worth investigating. Moreover, earlier research demonstrated that streptococci lacking a functional *galU* gene (encoding for UDPG:PP) were characterized by significantly reduced *in vitro* and *in vivo* virulence. Therefore, in this study we evaluated the anti-virulence activity of potential UDPG:PP inhibitors. They were selected *in silico* using a tailor-made streptococcal homology model, based on earlier listerial research. While the compounds didn’t affect bacterial growth, nor affected *in vitro* adhesion to and phagocytosis in macrophages, the amount of polysaccharide capsule was significantly reduced after co-incubation with these inhibitors. Moreover, co-incubation proved to have a positive effect on survival in an *in vivo Galleria mellonella* larval infection model. Therefore, rather than targeting bacterial survival directly, these compounds proved to have an effect on streptococcal virulence by lowering the amount of polysaccharide and thereby probably boosting recognition of this pathogen by the innate immune system. While the compounds need adaptation to broaden their activity to more streptococcal strains rather than being strain-specific, this study consolidates UDPG:PP as a potential novel drug target.

## Introduction

*Streptococcus pneumoniae* is one of the major causative agents of community acquired pneumonia and meningitis worldwide. Pneumonia is one the major causes of mortality in children under the age of five and is considered to be the third leading cause of death worldwide (Marangu and Zar, 2019; Peyrani et al., 2019). In 2015, it has been reported that 64% of child deaths due to pneumonia were caused by bacterial agents *S. pneumoniae* or *Haemophilus influenzae* (Marangu and Zar, 2019). Antibacterial treatment often consists of macrolides, amoxicillin, fluoroquinolones or cephalosporins (Peyrani et al., 2019). However, an increase in resistance toward macrolides has been reported (Yayan, 2014). For bacterial meningitis, *S. pneumoniae* is the causative pathogen in no less than 70% of all cases. While vaccination proved successful, there is now a re-emergence of pneumococcal infections due to serotype replacement, leading to incidences equal to the pre-vaccination era in some parts of Europe and North America (Koelman et al., 2019).

Amongst the variety of virulence factors the pneumococcus possesses, the polysaccharide capsule is considered as the most important one (Paton and Trappetti, 2019). Due to its presence, the first line of defense to pneumococcal invasion, i.e., macrophage phagocytosis, is limited and there is no adequate T-cell response (Geno et al., 2015). Deletion of this capsule drastically reduces pneumococcal virulence by increasing phagocytosis rates (MacLeod and Kraus, 1950; Preston and Dockrell, 2008). *In vitro*, non-encapsulated pneumococci show better adherence properties and also in *in vivo* nasopharyngeal colonization a decrease in capsule production is observed (Kadioglu et al., 2008; Gilley and Orihuela, 2014). However, when the transition from a commensal to an invasive lifestyle occurs, there is a clear upregulation of capsule production, likely due to the importance of it in evading the immune system (Kadioglu et al., 2008).

The most important gene locus for capsule production is the gene locus, which gives rise to over 90 different pneumococcal serotypes. Interestingly, these serotypes differ in the composition of the polysaccharide capsule, with a variety of sugars that can be included. A common feature in all serotypes is the presence of glucose (Glc) and/or galactose (Gal) (Geno et al., 2015; Paton and Trappetti, 2019). Apart from the *cps* gene locus, other genes are also known to be involved in the regulation of capsule production (Llull et al., 1999). One of these genes is the highly conserved *galU* gene, which encodes for uridine diphosphate glucose pyrophosphorylase (UDPG:PP, EC2.7.7.9). Briefly, UDPG:PP reversibly converts uridine diphosphate glucose (UDP-Glc) to glucose-1-phoshate (Glc-1-P) as part of the Glc and Gal metabolism. Furthermore, UDP-Glc is a key component in the formation of pneumococcal capsule (Mollerach et al., 1998). It has been shown that mutants lacking a functional *galU* gene do not form any detectable amount or at least show a significant downregulation of capsular polysaccharide (Mollerach et al., 1998; Cools et al., 2018). Moreover, *galU* mutants are more prone to *in vitro* macrophage phagocytosis and considerably less virulent *in vivo* (Cools et al., 2018). In addition, while UDPG:PP is present in almost all life on earth, prokaryotic UDPG:PPs are structurally unrelated to their eukaryotic counterparts (Flores-Díaz et al., 1997; Berbís et al., 2015). Also in other organisms, UDPG:PP alteration has been suggested as a way of battling infection, e.g., against *Escherichia coli, Klebsiella Pneumoniae*, and *Pseudomonas* (Berbís et al., 2015). We therefore postulate the UDPG:PP enzyme could present a potential effective drug target against *S. pneumoniae* as well.

As the crystal structure of pneumococcal UDPG:PP is currently unknown, it’s exact conformation and location of the binding site is unsure. Therefore, a recently published computational model based on listerial UDPG:PP was optimized for *S. pneumoniae* (Kuenemann et al., 2018). Adaptation of this model led to the identification of several hit compounds, which were characterized, predicted and selected *in silico* using 3D molecular docking in order to have a binding affinity that could result in some enzyme inhibitory activity. Three of these compounds were then evaluated in several *in vitro* and *in vivo* assays. Our main findings are that the tested potential inhibitors were indeed capable of modulating virulence. Moreover, this effect was dependent on the bacterial strain used, potentially enabling strain- and pathogen-specific virulence modulation. Therefore, more research should be done concerning these modulators in order to fully establish their bioprofiles and allow for a broader spectrum of inhibition in pneumococci. Overall, our data further establish UDPG:PP as a potential drug target against *S. pneumoniae* infections and confirm the significance of anti-virulence therapies as a promising avenue for fighting bacteria.

## Materials and Methods

### Homology Modeling and Protein Preparation

Homology models were built using Prime’s energy-based method included in the Schrödinger software suite based on D39/R6 and TIGR4 strains sequences (Jacobson et al., 2002, 2004). As template our in-house *Listeria monocytogenes* UDPG:PP 3D structure was used (Kuenemann et al., 2018). D39/R6 and TIGR4 share 98% of sequence identity based on ClustalW alignment (Supplementary Figure S1; Goujon et al., 2010). Meanwhile, these two *S. pneumoniae* strains share 63% of sequence identity and 78% of sequence similarity with *L. monocytogenes*. Once built, the models were standardized using the Protein Preparation Wizard from the Schrödinger Suite to ensure that there was no missing/clashing atom (including hydrogens). H-bonds assignment were performed at pH = 7 with PROPKA, and an additional energy minimization was performed with the OPLS3 force field (Harder et al., 2016). Obtained homology models were aligned on the listerial in-house structure, and Cα RMSD (Root Mean Square Deviation) were calculated. RMSD between the streptococcal strain D39/R6 homology model and *L. monocytogenes* is equal to 0.17 Å, whereas RMSD between streptococcal strain TIGR4 and *L. monocytogenes* is equal to 0.18 Å. The two *S. pneumoniae* homology models are available in the Supplementary Material and Supplementary Figure S2.

### Molecular Docking

Molecular docking of the 37 UDPG:PP ligands extracted from our previous study was done using both strains D39/R6 and TIGR4 homology models using Glide 2019-1 (Kuenemann et al., 2018). Extra precision (XP) mode with flexible ligand sampling was done following the same protocol as in our previous work (Friesner et al., 2004, 2006; Halgren et al., 2004; Kuenemann et al., 2018). Moreover, the same parameters were kept to generate the grid box using Receptor Grid Generation from Schrödinger. The outer box of 30 × 30 × 30 Å defines the volume in which the grid potentials are computed. The grid center has as coordinates *x* = 2.10, *y* = 46.44, and *z* = 14.85. The inner box of 10 × 10 × 10 Å represents the volume where the ligand center must be placed. The docking calculations allowed us to obtain, visualize and study the potential binding modes of the 37 listerial UDPG:PP inhibitors in the binding pockets for *S. pneumoniae* strains.

### Bacterial Strains, Cell Cultures and Compounds

All *S. pneumoniae* strains used in this study are listed in Table 1. Strains 85, 85 *galU* mutant, M23 and M23 *galU* mutant were a kind gift from Prof. Mollerach, Universidad de Buenos Aires, Argentina. Briefly, *galU* mutants were obtained through an interruption in the last 102 nucleotides of the gene leading to deletion of the last 33 C-terminal amino acids of the enzyme, which in turn leads to a disorganization of the enzyme tetramer (Mollerach et al., 1998; Martin et al., 2000; Cools et al., 2018). Reference strain TIGR4 (serotype 4) was obtained from ATCC^®^ (ATCC^®^ BAA-334^TM^). Reference strains D39 (serotype 2) and R6 (serotype 2^–^, unencapsulated) were obtained from NCTC^®^ (NCTC07466 and NCTC13276, respectively). Bacteria were cultured in brain-heart infusion (BHI) broth (LabM) or on 5% sheep blood agar plates (Tryptic Soy Agar, LabM, Oxoid) at 37°C and 5% CO_2_. Murine macrophage cells were obtained from ATCC^®^ (RAW 264.7, ATCC^®^ TIB-71^TM^) and grown in Dulbecco’s modified Eagle’s medium (DMEM) supplemented with 10% inactivated fetal calf serum (IFCS) and 1% pyruvate (all from Sigma-Aldrich) under the same conditions. Three compounds were purchased from Asinex Corporation (BDH 33910157, BDG 33920985, BDH 33910188). Compound structures, properties and codes provided by the vendor are listed in Table 2. Compounds were suspended upon arrival in dimethyl sulfoxide (DMSO) (Novolab) to 10 mM and stored in the dark at room temperature.

**TABLE 1:** *S. pneumoniae* strains used in this study.

***S. pneumoniae* strain**	**Serotype**	**Source**
85	Serotype 14	Prof. Mollerach, Universidad de Buenos Aires, Argentina
85 *galU* mutant	Serotype 14, *galU* mutated	Prof. Mollerach, Universidad de Buenos Aires, Argentina
M23	Serotype 3	Prof. Mollerach, Universidad de Buenos Aires, Argentina
M23 *galU* mutant	Serotype 3, *galU* mutated	Prof. Mollerach, Universidad de Buenos Aires, Argentina
TIGR4	Serotype 4	ATCC^®^, BAA-334
D39	Serotype 2	NCTC^®^, NCTC07466
R6	Derived from serotype 2, capsule deficient	NCTC^®^, NCTC13276

**TABLE 2:** Compounds used in the *in vitro* and *in vivo* assays.

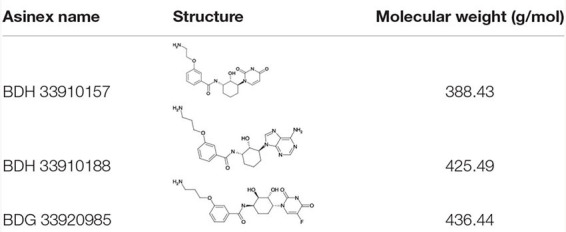

*All compounds were purchased from Asinex Corporation and were resuspended in DMSO to a stock concentration of 10 mM.*

### Planktonic Growth

Planktonic growth curves were obtained over an 8-h period. All strains were grown in BHI broth at 37°C and 5% CO_2_, as advised by ATCC^®^, with or without 50 μM of compound. At 2-h intervals, the concentration was determined by viable plate count. For each strain and compound, 3 independent repeats were carried out.

### Antibiotic Activity

Minimal inhibitory concentrations (MIC) of all compounds were determined using a resazurin assay as described previously (Torfs et al., 2018). Briefly, 100 μL of a ½ serial dilution series of compounds in BHI broth was added to 96-well plates, after which bacteria were added to a final concentration of 2.5 × 10^5^ CU/mL in 200 μL. The highest concentration of compounds was 64 μM after addition of bacteria. After 20 h of incubation at 37°C and 5% CO_2_, 20 μL of 0.005% resazurin was added. After an additional incubation of 90 min, fluorescence was measured at λ_*emission*_ = 590 nm, λ_*excitation*_ = 550 nm using a spectrophotometer (Promega Discover). For each strain, 2 independent repeats were carried out.

### Cytotoxicity Assay

MRC-5 cells were grown into polystyrene 96-well plates at an initial concentration of 1.5 × 10^5^ cells/mL cells per well and incubated at 37°C and 5% CO_2_. In each well, 190 μL of cell suspension was added together with 10 μL of watery compound dilutions. Cell growth was compared to untreated control wells (100% cell growth) and medium-control wells (0% cell growth). After 3 days of incubation, cell viability was assessed using resazurin as described earlier. A compound is classified non-toxic when the IC_50_ is greater than 20 μM. Tamoxifen was used as a positive control.

### Macrophage Assay

RAW 264.7 cells were seeded into polystyrene 24-well plates at 2 × 10^5^ cells per well and incubated at 37°C and 5% CO_2_, 24 h prior to infection. Bacteria were grown as described earlier for 4 h prior to infection, with or without 50 μM of compound. Then, bacteria were added to cells at a multiplicity of infection (MOI) of 10 in DMEM + 10% iFCS + 1% pyruvate as described previously (Domon et al., 2016). Plates were incubated for 90 min at 37°C and 5% CO_2_. Cells were washed twice with PBS/Ca^2+^Mg^2+^, to wash away all loose bacteria. For determination of intracellular bacteria, 50 mg/mL gentamicin (Life Technologies) was added at 200 μL/mL in DMEM + 10% iFCS + 1% pyruvate. Cells were incubated for 60 min at 37°C and 5% CO_2_ to kill all extracellular bacteria. Afterward, cells were lysed using 200 μL 0.1% Triton X-100 (Sigma-Aldrich) for 10 min at room temperature and the concentration of internal bacteria was determined using the viable plate count method. To determine the total amount of intracellular and adhered bacteria, 200 μL 0.1% Triton X-100 was added directly after washing the cells and the concentration was determined by viable plate count. For each strain, three independent repeats were carried out.

### FITC-Dextran Exclusion Assay

The degree of encapsulation was determined by measuring the zone of exclusion of FITC-dextran (200 kDa, Sigma-Aldrich), as described previously (Gates et al., 2004; Weinberger et al., 2009; Cools et al., 2018). Briefly, bacteria were grown as described earlier until logarithmic phase with or without 50 μM of compound, centrifuged for 5 min at 5000 *g* and resuspended in PBS. To 100 μL of bacterial suspension 20 μL FITC-dextran (10 μg/mL in water) was added and 10 μL of this suspension was subsequently used to create wet mounts with cover slips. The slides were viewed on a Zeiss Observer.Z1 microscope with a 100x objective, and fluorescent images were captured with a Zeiss AxioCam MRm camera. The images were converted into grayscale and analyzed using ImageJ software. The area of FITC exclusion was determined. For each fluorescent image, a brightfield image was also recorded in order to count the number of bacteria per chain. For each strain, the mean of 150–300 cells was determined, and at least two images were collected from each of at least two independently prepared slides.

### *Galleria mellonella* Killing Assay

Larvae were purchased from a local vendor (Anaconda Reptiles, Kontich, Belgium) and stored in wood chips at 15°C before use. Four hours before use, larvae were put at 4°C. A sterile 20 μL Hamilton syringe was used to inject 10 μL aliquots of bacterial suspensions into the hindmost left proleg of *Galleria mellonella*. Bacteria were grown mid logarithmic phase for 6 h with or without 1, 10, 50 or 200 μM compounds, washed and resuspended in PBS before infection. The control group was injected with 10 μL PBS. A minimum of 10 larvae per group was used. Following the injections, larvae were incubated at 37°C in the dark for several days to allow progression of the pneumococcal infection. Every 24 h, larvae were scored as dead or alive. Larvae were determined dead when no signs of movement could be observed in response to external stimuli. For each strain, at least four independent repeats were carried out.

### Statistical Analysis

Data were analyzed for statistical significance using Graphpad Prism Version 8. Continuous variables were compared by one-way Anova, two-way Anova, *t*-test or survival analyses. Statistical significance was defined as *P* < 0.05. Statistical significance between groups is mentioned as asterisks in figures (^∗^ *p* ≤ 0.05; ^∗∗^ *p* ≤ 0.01; ^∗∗∗^ *p* ≤ 0.001; ^****^ *p* ≤ 0.0001).

## Results

Molecular docking of the 37 *listerial* UDPG:PP hit compounds from our previous study (Kuenemann et al., 2018) was performed toward the newly built strains D39/R6 and TIGR4 UDPG:PP homology models, as described in the “Materials and Methods” section. First, the reproducibility of our docking results regarding listerial UDPG:PP using the newest version of 2019 Glide was tested. While some minor differences in the docking scores (DS) obtained by the best poses were observed, overall the most potent compounds (BDG 33920985, BDF 34002917, BDH 33911533, and BDH 34012219) were still ranked in the top 5 (Table 3). This rarely reported reproducibility test was critical for ensuring the validity of the following docking calculations.

**TABLE 3:** Docking scores (DS) in kcal/mol for top five ranked compounds.

	**Model**	**Rank 1**	**Rank 2**	**Rank 3**	**Rank 4**	**Rank 5**
*S. pneumoniae*	D39/R6	**BDH 33911485** (−8.92)	**BDH 34000291** (−8.37)	**BDH 33910188** (−8.35)	BDH 33911472 (−8.30)	BDG 33920985 (−8.27)
	TIGR4	BDF 34002917 (−9.31)	**BDH 33911485** (−9.17)	**BDH 34000291** (−9.06)	**BDH 33910188** (−8.46)	BDH 33920962 (−7.58)
*L. monocytogenes*	In-House redocking 2019	BDG 33920985 (−9.93)	BDF 34002917 (−9.29)	BDH 33911533 (−8.86)	BDH 34012219 (−9.03)	BDH 34012595 (−8.39)
	Kuenemann et al., 2018	BDG 33920985 (−10.02)	BDH 33910157 (−9.59)	BDF 34002917 (−9.20)	DH 33911533 (−9.16)	BDH 34012219 (−9.03)

*In bold are compounds ranked as top five for both *Streptococcus pneumoniae* strains.*

Regarding the docking for streptococcal UDPG:PP, most of the resulting DS were found to be in the same binding affinity range. There were a few exceptions: for instance, we noted that BDH 33910157 obtained a very good DS for listerial UDPG:PP but not at all for *S. pneumoniae* models (Supplementary Table S1). The compounds achieving the best DS (thus lowest values) were BDH 33911485, BDH 33910188, BDF 34002917, BDH 34000291, and BDG 33920985.

**FIGURE 1:**
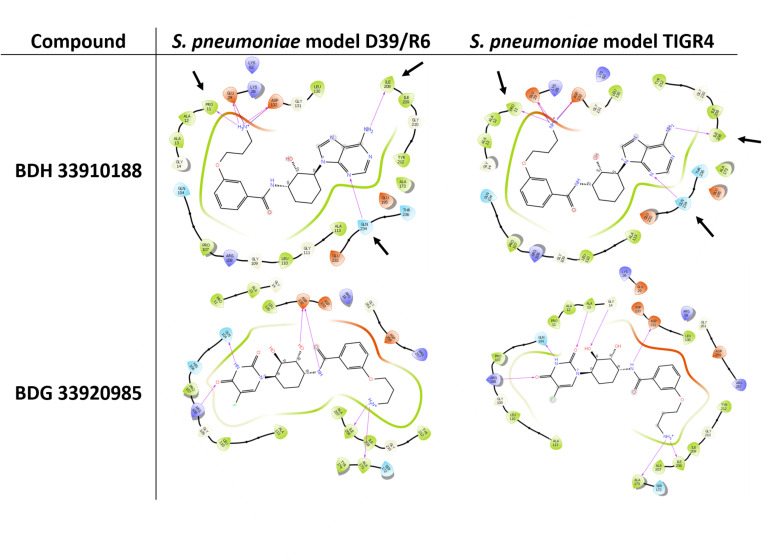
Illustration of protein-ligand interaction. Compounds BDH 33910188 and BDG 33920985 with UDPG:PP of streptococcal strains D39/R6 and TIGR4. Arrows point toward the residues which could be responsible for the improvement in the docking score (DS) in the model. For compound BDH 33920985, DS of *S. pneumoniae* models were lower than those of the *L. monocytogenes* models.

Three compounds were selected to perform the experimental study according to their DS and to their availability in Asinex stock, i.e., BDG 33920985 (active on *L. monocytogenes* and predicted to be active toward D39/R6), BDH 33910157 (only active on *L. monocytogenes*, as negative control) and BDH 33910188 (predicted to be active on D39/R6 and TIGR4). Their protein-ligand interactions were studied and the associated residues are reported in Supplementary Figure S3. Several residues including Pro11, Glu29, Arg108, Asp132, Ile208, and Gln234 are predicted to be critical for the binding mode of the compounds (Figure 1). For instance, Asp132 is predicted to establish a strong H-bond with the terminal amine moiety of BDH 33910188 and the amide group of BDG 33920985. Similarly, Arg108 could be of importance for the anchoring of BDG 33920985.

### *In vitro* Bacterial Growth Is Unaffected

**FIGURE 2:**
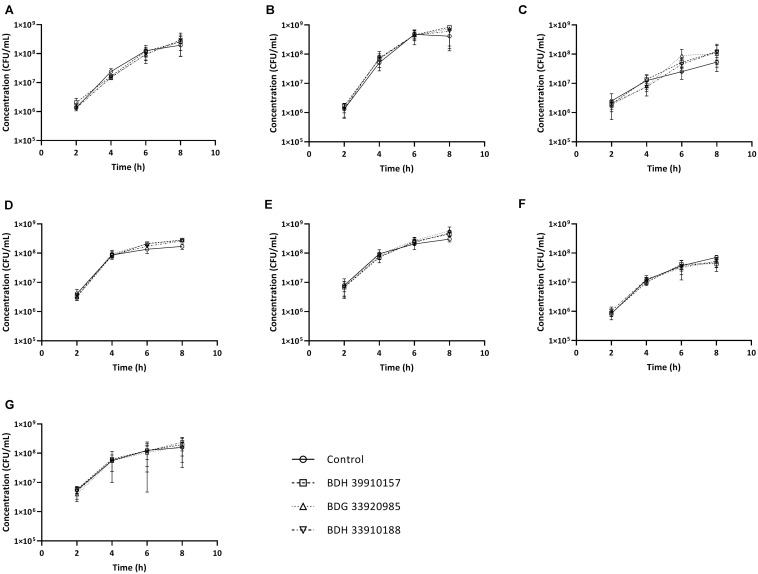
Planktonic growth curves for *S. pneumoniae* strains in presence or absence of 50 μM of compound. **(A)** Strain TIGR4, **(B)** Strain D39, **(C)** Strain R6, **(D)** Strain 85, **(E)** Strain 85 *galU* mutant, **(F)** Strain M23, **(G)** Strain M23 *galU* mutant. Error bars represent SD. No statistical differences between control groups and treatment groups were observed (two-way Anova) (*n* = 3).

Since UDPG:PP is part of the glucose and galactose metabolism, inhibition of this enzyme could potentially lead to changes in growth characteristics. To assess whether addition of the compounds to growing bacterial cultures had an effect on pneumococcal viability, planktonic growth in the presence of these compounds was evaluated. As seen in Figure 2, addition of the compounds didn’t alter planktonic pneumococcal growth for any pneumococcal strain (*p* > 0.05, Two-way Anova). Also, antimicrobial properties of the compounds were evaluated using a standard antimicrobial susceptibility test. Again, even the highest tested concentration of compounds, 64 μM, didn’t result in a decrease in viability. Taken together, these results indicate the potential inhibitors have no effect on pneumococcal viability or survival, which in light of the ongoing battle against antimicrobial resistance, is an important feature. Lastly, cytotoxicity of the compounds to MRC-5 cells was determined. Compounds BDH 33910157 and BDH 33010188 showed an CC_50_ over the maximal tested concentration of 64 μM, for compound BDG 33920985 the CC_50_ was 43.7 μM. As compounds are considered cytotoxic when the CC_50_ is below 20 μM, none of the compounds showed cytotoxic activity.

### Capsule Production Is Lowered but *in vitro* Phagocytosis Remains Unaltered

**FIGURE 3:**
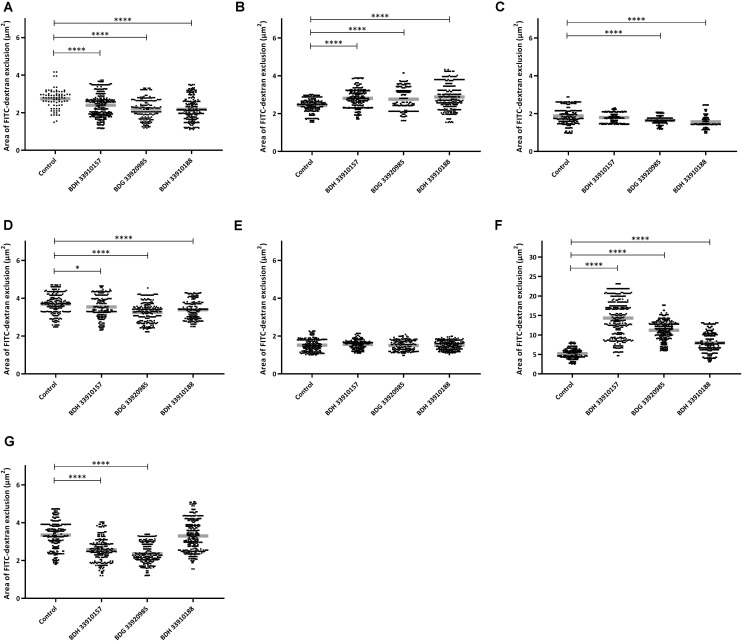
FITC-dextran exclusion assay. Area of exclusion (μm^2^) in presence or absence of 50 μM of compound. **(A)** Strain TIGR4, **(B)** Strain D39, **(C)** Strain R6, **(D)** Strain 85, **(E)** Strain 85 *galU* mutant, **(F)** Strain M23, **(G)** Strain M23 *galU* mutant. Error bars represent SD. Asterisks represent statistical differences between control group and treatment groups (Unpaired *t*-test; * *p* ≤ 0.05; **** *p* ≤ 0.0001) (*n* = 150–300).

**FIGURE 4:**
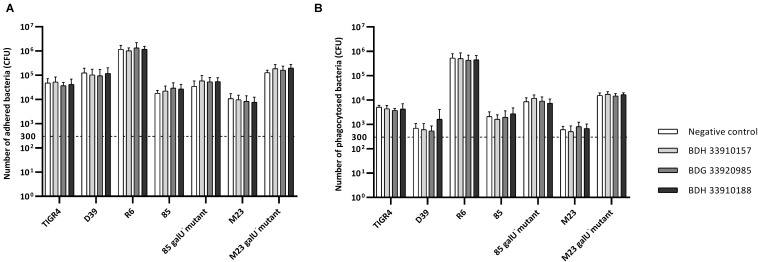
Number of adhered and phagocytosed bacteria with RAW 364.7 macrophage cells. **(A)** Total number of adhered and phagocytosed bacteria, obtained with Triton X-100 treatment, **(B)** Number of phagocytosed bacteria, obtained after removal of extracellular bacteria by gentamicin treatment. No statistical differences between control groups and treatment groups were observed (Two-way Anova) (*n* = 3 × 2).

To evaluate the effect of the compounds on the amount of capsule produced by *S. pneumoniae*, the bacteria were measured using the FITC-dextran exclusion assay. This assay measures the size of the bacteria, including their capsule. While a polysaccharide capsule is not visible using a regular brightfield light microscope, fluorescence microscopy can be used. As fluorescently labeled dextrans are unable to pass the polysaccharide barrier, the size of fluorescent exclusion can be directly linked to the size of the bacteria and thus to the amount of polysaccharide capsule. The compounds slightly, but significantly, lower the size of streptococcal strains TIGR4, R6, 85 and the *galU* mutant of strain M23 (Figures 3A,C,D,G) (*p* < 0.0001 for all combinations, except strain 85 – BDH 33910157: *p* = 0.0297, One-way Anova). Only compounds BDH 33910157 and BDH 33910188 had no effect on the size of strains R6 and M23 *galU* mutant, respectively (strain R6 – BDH 33910157, *p* = 0.0709, strain M23 *galU* mutant – BDH 33910188: *p* = 0.8351, One-way Anova). At least for strains R6 and M23 *galU* mutant, which were both already capsule deficient prior to co-incubation with compounds, this implies another mechanism of action of these compounds where e.g., also the cell wall of the bacteria or the glucose metabolism is involved (Berbís et al., 2015). However, not all capsule deficient strains show an additional decrease in size. The compounds had no effect on the size of strain 85 *galU* mutant (Figure 3E) (strain 85 *galU* mutant – BDH 33910157: *p* = 0.3378, strain 85 *galU* mutant – BDG 33920985: *p* = 0.9701, strain 85 *galU* mutant – BDH 33910188: *p* = 0.9760, One-way Anova). Lastly, in strains D39 and M23 the compounds had an opposite effect, as co-incubation with compounds increased the area of exclusion, thus increased bacterial size (Figures 3B,F) (*p* < 0.0001 for all combinations, One-way Anova). In strain D39 these effects are rather limited, but in strain M23 – which is the largest of all pneumococcal strains used in this study – the compounds have a profound effect on bacterial size. Off topic, capsule production was significantly lower in *galU* mutant strains 85 and M23 compared to their respective parent strains (*p* < 0.0001 in both cases, Unpaired *t*-test). As the polysaccharide capsule is the most predominant factor in macrophage adhesion and phagocytosis, differences in bacterial size are postulated to lead to differences in cellular interactions. As reported before, *galU* mutated strains clearly show an increase in macrophage phagocytosis compared to their non-mutated parent strains (strain M23 – strain M23 *galU* mutant: *p* < 0.0001, strain 85 – strain 85 *galU* mutant: *p* = 0.0004, Unpaired *t*-test) (Figure 4; Cools et al., 2018). However, addition of the compounds rendered no changes in phagocytosis rates. Even for strain M23, where the largest variation in size was recorded, no change in adherence or phagocytosis was seen. This implies that either the changes in polysaccharide production are not diverse enough to provoke changes or that the assay is not sensitive enough to pick up on them.

### *In vivo* Virulence of Pneumococci Is Attenuated

**FIGURE 5:**
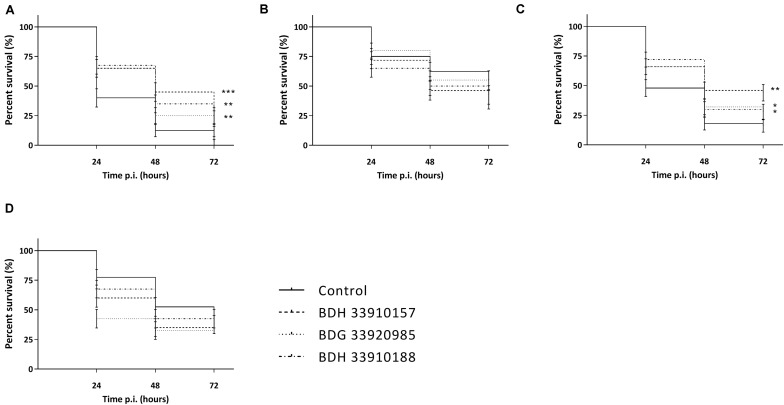
Kaplan-Meier survival curves of *G. mellonella* after infection with *S. pneumoniae* grown in presence or absence of compounds. **(A)** Strain TIGR4, **(B)** Strain D39, **(C)** Strain 85, **(D)** Strain M23. Error bards represent SE. Asterisks represent statistical differences between control group and treatment groups (Survival analysis; * *p* ≤ 0.05, ** *p* ≤ 0.01, *** *p* ≤ 0.001) (*n* = 4 × 10).

**FIGURE 6:**
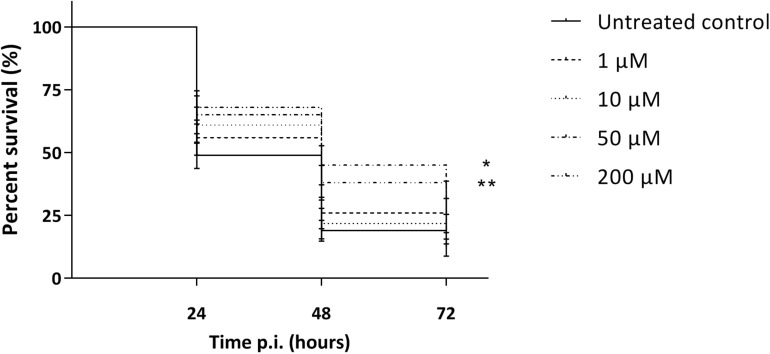
Kaplan-Meier survival curves of *G. mellonella* after infection with *S. pneumoniae* strain TIGR4 grown in presence of 1, 10, 50 or 200 μM of compound BDH 33910157. Error bards represent SE. Asterisks represent statistical differences between control group and treatment groups (Survival analysis; * *p* ≤ 0.05, ** *p* ≤ 0.01) (*n* = 5 × 10).

As the macrophage assay proved no changes in macrophage functionality, a difference in *in vivo* virulence was not expected. In order to assess virulence of all virulent pneumococcal strains (TIGR4, D39, M23, and 85) and the effect of addition of compounds, a *G. mellonella* infection model was used. This model is easy to use, cheap and it is possible to set up large experiments including several variables (Cools et al., 2019). Contrary to prior expectations, there was an effect of the compounds in several virulent pneumococcal strains (Figure 5). Co-incubation with compounds lead to a decrease in virulence for strains TIGR4 and 85 (Figures 5A,C) (strain TIGR4 – BDH 33910157: *p* = 0.0005, strain TIGR4 – BDG 33920985: *p* = 0.0193, strain TIGR4 – BDH 33910188: *p* = 0.0016, strain 85 – BDH 33910157: *p* = 0.00330, strain 85 – BDG 33920985: *p* = 0.0640, strain 85 – BDH 33910188: *p* = 0.0211, Log-rank Mantel-Cox test). This effect coincides with the data of the FITC-dextran exclusion assay, where the compounds were able to reduce bacterial size also in these strains. For strains D39 and M23, where the compounds were not able to reduce bacterial size, the virulence was also not altered (Figures 5B,D) (strain D39: *p* = 0.3428, strain M23: *p* = 0.3917, Log-rank mantel-Cox test). Importantly, the increase in bacterial size seen in these strains didn’t render them more pathogenic *in vivo*. Lastly, a dose-response curve was obtained for the most active compound, BDH 33910157 (Figure 6). Lowering the dose to 1 or 10 μM rendered the compound inactive, while increasing it to 50 or 200 μM significantly improved larval survival compared to an uninfected control group (50 μM: *p* = 0.0173, 200 μM: *p* = 0.0033, Log-rand Mantel-Cox test).

## Discussion

While pneumococcal vaccination has proven to be beneficial and has led to a decrease in morbidity and mortality, there are several downsides. Most importantly, in the years after the introduction of the vaccine a serotype switch to non-vaccine serotypes has been observed (Van Der Linden et al., 2015). Furthermore, herd immunity is only present in young children and not observed in non-vaccine serotypes (Berical et al., 2016; Isturiz et al., 2017). On the other hand, treatment against pneumococcal infections mostly consists of amoxicillin, vancomycin, moxifloxacin or cefuroxime, depending on the antimicrobial susceptibility profile of the infecting agent (Peyrani et al., 2019). While commonly at least one of these antibiotics is capable of battling the pneumococcal infection, antimicrobial resistance is on the rise worldwide and the use of antibiotics should be limited at all cost (McEwen and Collignon, 2018). Therefore, there is a need for novel innovative ways of battling pneumococcal infections, diverging from standard antimicrobial molecules or vaccination strategies. In this study, inhibition of UDPG:PP is proposed and researched as such a novel approach. UDPG:PP is a commonly found enzyme in most life forms, being present in both eukaryotes and prokaryotes (Flores-Díaz et al., 1997; Berbís et al., 2015). Within these domains, UDPG:PP serves a multitude of functions, based on the conversion of UDPG-Glc to Glc-1-P (Mollerach et al., 1998). As glucose plays a pivotal role in a variety of cell processes, UDPG:PP is found to play a role in the integrity of cell membranes, functionality of flagellae, production of LPS and the pneumococcal polysaccharide capsule (Komeda et al., 1977; Deng et al., 2010). While UDPG:PP has previously been proposed as a valid novel drug target, up until recently no inhibitors were known (Kuenemann et al., 2018). In 2018, UDPG:PP inhibitors against listerial UDPG:PP were screened using an *in silico* modeling approach, leading to the identification of several compounds with anti-listerial activity. In *L. monocytogenes*, UDPG:PP is important in the first step of wall teichoic acid galactosylation, which renders the use of antibiotic cefotaxime useless. Addition of UDPG:PP inhibitors elevated the MIC values of this antibiotic drastically, thereby proving UDPG:PP was effectively inhibited (Kuenemann et al., 2018).

In this study, a similar approach to identify pneumococcal UDPG:PP inhibitors was conducted. As the crystal structure of pneumococcal UDPG:PP is currently unknown, the genome of two *S. pneumoniae* strains, TIGR4 and D39, served as a template for the computational part. According to the *in silico* modeling compound BDH 33910157 showed the lowest binding affinity toward *S. pneumoniae* strains TIGR4 and D39. Compound BDG 33920985 only showed a good binding affinity toward strain D39, while BDH 33910188 showed a good binding affinity toward both strains. However, in most biological assays there were no differences between the compounds. Also, while the genome of strains TIGR4 and D39 were both used for the *in silico* modeling, none of the compounds had an effect on strain D39 in a biological setting. To the contrary, a significant increase in bacterial size of strain D39 was seen after incubation with these compounds. This implies *in silico* modeling was effective but not 100% accurate. However, modeling was performed solely based on the primary sequences of these strains without crystal structures of the target. This could explain the contradicting results when using other pneumococcal strains, as small genomic differences could lead to e.g., a significant change in the three-dimensional conformation of the enzyme, increase or decrease in binding affinity, shielding of the binding place. I*n silico* modeling without an actual crystal structure being available is challenging (Kuenemann et al., 2018). While the pneumococcal enzyme has been purified before, the crystal structure remains unidentified (Zavala et al., 2017). However, elucidation of this structure could greatly improve *in silico* modeling and development of novel inhibitors. Currently, the crystal structure of UDPG:PP is only known for several eukaryotes and following bacteria: *Helicobacter pylori* (PDB codes 3JUJ and 3JUK) (Kim et al., 2010), *E. coli* (PDB code 2E3D) (Thoden and Holden, 2007a), *Corynebacterium glutamicum* (PDB code 2PA4) (Thoden and Holden, 2007b), *Acinetobacter baumannii* (PDB codes 6IKX and 6IKZ) (Lee and Kang, 2019), *Sphingomonas elodea* (PDB 2UX8) (Aragao et al., 2007), *Erwinia amylovora* (PDB code 4D48) (Benini et al., 2017), *Yersinia pestis* (PDB code 6MNU) (Gibbs et al., 2019), and *Burkholderia spp.* (PDB codes 5VCT, 5VE7, 5J49, 5I1F) (Abendroth et al., 2016a,b, 2017a,b). Apart from the aforementioned issues with *in silico* modeling based on genetic sequences, biological effects could be lower than expected due to marginal uptake in the cells, rather than poor enzymatic binding. However, in previous research these compounds have proven to be effective against closely related *L. monocytogenes*, implying uptake is possible (Kuenemann et al., 2018).

Several lead compounds were identified *in silico* and a selection of three compounds was tested in subsequent *in vitro* and *in vivo* biological assays. The *G. mellonella* larval *in vivo* model possesses only an innate immune system. This model allowed a better study of the first line of defense and primary recognition, without the interference of an adaptive immunity compared to more complex vertebrate models (Tsai et al., 2016). Overall, the effect of the compounds on bacterial size thus amount of polysaccharide capsule, as seen in the FITC-dextran exclusion assay, was rather limited. While a mutation of UDPG:PP, leading to a dysfunctional enzyme, showed a decrease of approximately 50% in overall size, addition of the compounds decreased the bacterial size by only 10–20%. However, virulent bacterial strains TIGR4 and 85, that showed a statistical decrease in bacterial size – even though small – also showed a clear decrease in *in vivo* virulence. This implies the compounds were capable of inhibiting the UDPG:PP enzyme, which led to at least a partial decrease in pneumococcal polysaccharide capsule and consecutively to a better recognition of the pathogen by the innate immune system. Strains D39 and M23, for which no or an adverse effect of the compounds on bacterial size was observed, showed no differences in *in vivo* virulence. While for strain M23 the polysaccharide capsule size greatly increased (approximately 200%), the innate immune system was able to withstand the infection equally as it did not lead to an increase in *in vivo* virulence. To explain the different effects on bacterial size, the biochemical structure of each serotype provides more insight. Both strains D39 and M23 (respectively, serotypes 2 and 3) incorporate glucuronic acid (GlcA) in their capsules (Geno et al., 2015). UDP-GlcA is readily formed out of UDP-Glc, regulated by UDPG:PP (Mollerach et al., 1998). A decrease in available UDP-Glc and subsequent UDP-GlcA might lead to a less well-organized capsule, looser conformation and thus increase in overall size. On the other hand, the capsule of strains TIGR4 and 85 (respectively, serotype 4 and 14) doesn’t contain GlcA (Geno et al., 2015). Therefore, a shortage in UDP-Glc won’t directly influence their capsule but will lead to a general shortage in sugars. On its turn, this could lead to a decrease in overall capsule production. As the compounds don’t completely inhibit UDPG:PP, there is no complete abolishment of capsule. If UDPG:PP would be fully inhibited, the shortage in UDP-Glc would be more severe, which would render the bacteria unable to synthetize capsule regardless of serotype, as was seen earlier when using *galU* mutant strains (Cools et al., 2018). It should be noted that bacterial size was also affected in some strains already deficient either of capsule (R6) or of functional UDPG:PP (M23 *galU* mutant). As UDPG:PP serves a multitude of purposes in the prokaryotic cell, it is feasible that another pathway was also affected, especially when there is no capsule present (Berbís et al., 2015). On the other hand, the compounds were not screened for their selectivity against UDPG:PP, thus they might potentially interfere with other enzymes as well, which could also have an effect on capsule formation.

Furthermore, there was no effect of the compounds on planktonic pneumococcal growth nor could a MIC be determined, thus the decrease in virulence could not be attributed to a classic antimicrobial working mechanism. This implies the bacteria encountered no immediate negative consequences of these compounds. More likely, the larval innate immune system got triggered by the decreased amount of capsule, which led to an increase in phagocytosis rate and decrease in virulence, as observed before (Preston and Dockrell, 2008). This sort of therapy is considered a good adjuvant to the conventional antimicrobial therapy in the light of the battle against antimicrobial resistance (Sadgrove and Jones, 2019). Lastly, while the *in vivo* model rendered multiple statistical differences between treated groups and untreated controls, this effect was not seen in *in vitro* adherence to and phagocytosis in a macrophage cell line. However, pneumococcal strains without a functional UDPG:PP enzyme clearly showed an increase in adherence and phagocytosis compared to their non-mutated parent strains (Cools et al., 2018). This again implies that, while the compounds might partially inhibit the enzyme, they are not capable of fully inhibiting it, thus abolishing all polysaccharide capsule. The *in vitro* macrophage assay is probably not sensitive enough to detect these more subtle differences in virulence. Off note, this finding stretches the importance of fast and cheap *in vivo* models, to consolidate or contest *in vitro* data before either discarding compounds or research ideas or moving toward more complex *in vivo* models (Cools et al., 2019).

Several other anti-virulence drug targets against pneumococci have been proposed. Concerning inhibition of polysaccharide capsule, CpsB, a tyrosine phosphatase encoded by *cpsB*, has been suggested as novel drug target, as *cpsB* mutants have been shown to be avirulent in several animal models of infection (Morona et al., 2004; Standish et al., 2012; Monteiro Pedroso et al., 2017). Fascioquinol E – an extract derived from the marine sponge *Fasciospongia spp.*, has been shown to inhibit CpsB phosphatase activity and to increase macrophage adherence *in vitro* (Standish et al., 2012). Other strategies include modification of the bacterial cell wall, inhibition of pneumolysin and inhibition of quorum sensing. Lysozyme, a component of the human immune system, is known to be important in degradation of bacterial peptidoglycan layers, thereby destabilizing the bacterial cell wall. However, pneumococci can withstand this lysing enzyme by a deacetylation process, catalyzed by peptidoglycan N-acetylglucosamine deacetylase A (PgdA). Mutant pneumococci lacking this enzyme are more susceptible to lysozyme *in vitro* and show a reduction in virulence *in vivo* (Vollmer and Tomasz, 2002). Also, *in silico* inhibitors of PgdA have been described (Bui et al., 2011). Multiple studies have shown that inhibition of pneumolysin, a virulence factors known to be essential for bacterial survival in the respiratory tract, reduces mortality in *in vivo* models (Kadioglu et al., 2008; Arzanlou et al., 2011; Li et al., 2015; Zhao et al., 2016, 2017; Song et al., 2017a, b). Apart from direct inhibition, also sequestration of pneumolysin in liposomes has been shown beneficial on infection outcome in animal models (Henry et al., 2015; Baumgartner et al., 2016). Several antimicrobial peptides, analogs of indolicidin and ranalexin, are also proposed as pneumolysin inhibitors. However, their mechanism of action might also include inhibition of autolysin (Jindal et al., 2015, 2017). Finally, quorum sensing inhibitors have proven to effectively prevent *in vitro* and/or *in vivo* biofilm formation on middle ear implants and migration of pneumococci to the blood (Yadav et al., 2012, 2014, 2015; Cevizci et al., 2015; Motib et al., 2017).

In conclusion, we have shown that UDPG:PP inhibitors possess a great potential in the search for novel anti-virulence modulators. However, elucidating the crystal structure of pneumococcal UDPG:PP would benefit the development of adequate and selective inhibitors. Furthermore, development of future inhibitors should focus on inhibiting pneumococcal UDPG:PP regardless of *S. pneumoniae* serotype, while disregarding other prokaryotic or eukaryotic UDPG:PP. This research is the first report on using UDPG:PP inhibitors against pneumococcal infections and supports the idea of using UDPG:PP as a novel drug target.

## Data Availability Statement

The datasets generated for this study are available on request to the corresponding author.

## Author Contributions

FC, PC, and DF conceived the presented idea. FC, NG, and DT carried out the experiments. DT, PD, DF, and PC contributed to interpretation of results. FC and DF wrote the manuscript with support of DT, NG, PD, and PC. DF and PC helped supervise the project. All authors contributed to manuscript revision, read and approved the submitted version.

## Conflict of Interest

The authors declare that the research was conducted in the absence of any commercial or financial relationships that could be construed as a potential conflict of interest.
