# Supplementary material for: In vitro and in vivo Evaluation of in silico Predicted Pneumococcal UDPG:PP Inhibitors
Source: Front Microbiol. 2020 Jul 15;11:1596. doi: 10.3389/fmicb.2020.01596 (PMC7373766; doi:10.3389/fmicb.2020.01596)
Supplement: Supplementary file 1 [file Data_Sheet_1.docx]

**Supplementary material**

***In vitro* and *in vivo* evaluation of *in silico* predicted pneumococcal UDPG:PP inhibitors**

**Cools F^1^, Triki D^2^, Geerts N^1^, Delputte P^1^, Fourches D^2^, and Cos P^1,*^**

^1^Laboratory for Microbiology, Parasitology and Hygiene (LMPH), University of Antwerp, Universiteitsplein 1, 2610 Wilrijk, Belgium

^2^Department of Chemistry, Bioinformatics Research Center, North Carolina State University, Raleigh, USA.

*** Correspondence:**Paul Cos
paul.cos@uantwerpen.be

**Keywords**: ***Streptococcus pneumoniae*, GalU, *in silico* modeling, virulence, *Galleria mellonella,* novel drug target**


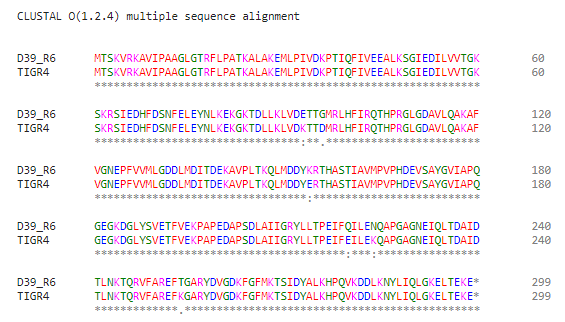


**Supplementary Figure S1:** Alignment of Streptococcus pneumoniae strains D39/R6 and TIGR4 using ClustalW (Goujon et al., 2010).


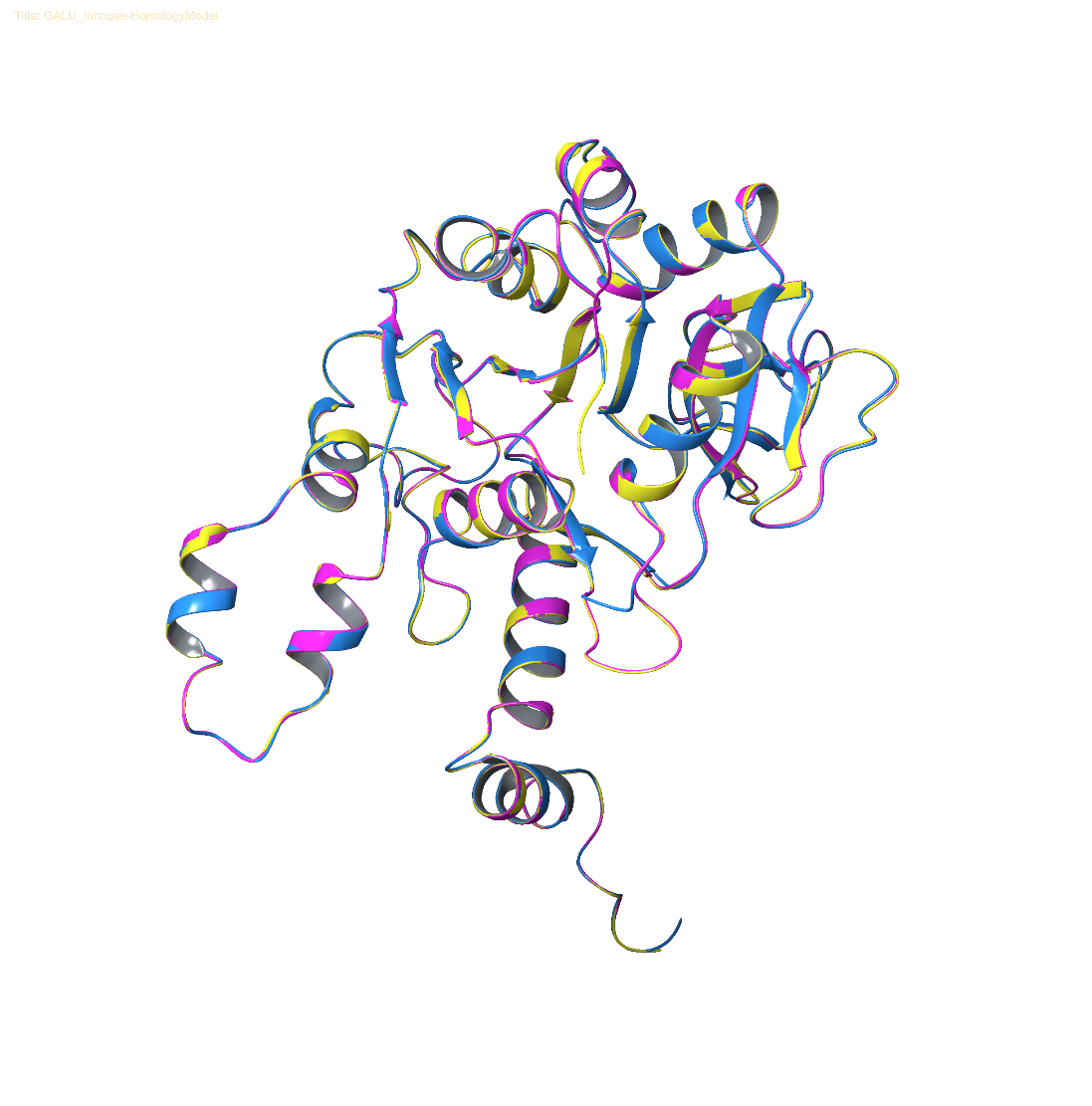


**Supplementary Figure S2:** Alignment of homology models. The in-house Listeria monocytogenes is in drawn blue, Streptococcus pneumoniae strain D39/R6 is drawn in yellow and S. pneumoniae strain TIGR4 is drawn in magenta.

**
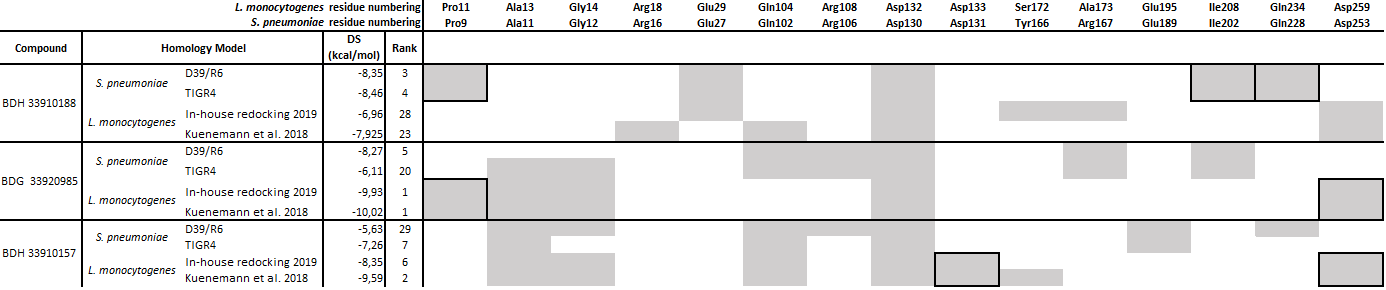
**

**Supplementary Figure S3:** **Interactions of the three selected ligands for the experimental study**. Grey cells represent the presence of a non-covalent interactions; cells are empty when there was no interaction observed. Cells are bordered when a residue could improve the docking score (DS) in a model. E.g.: DS of BDH 33910188 is the best in streptococcal strains D39/R6 and TIGR4 comparing to L. monocytogenes. It could be increased through the interaction of Pro11, Ile208, and Gln234.

**Supplementary Table S1:** Docking scores (DS) for the 37 selected compounds in Streptococcus pneumoniae and our in-house Listeria monocytogenes homology models. Top 5 ligands are in bold. DS are in grey is when the DS difference between published results from Kuenemann et al., 2018 and the In-house redocking 2019 is > 1 kcal/mol.

|  | **Homology model** | | | | | | | |
| --- | --- | --- | --- | --- | --- | --- | --- | --- |
|  | ***S. pneumoniae*** | | | | ***L. monocytogenes*** | | | |
|  | **D39/R6** | | **TIGR4** | | **In-house redocking 2019** | | **Kuenemann et al. 2018** | |
| **Compound** | **DS (kcal/mol)** | **Rank** | **DS (kcal/mol)** | **Rank** | **DS (kcal/mol)** | **Rank** | **DS (kcal/mol)** | **Rank** |
| BDF 33746139 | -6.48 | 17 | -7.19 | 12 | -8.19 | 9 | -8.31 | 15 |
| BDG 33920985 | **-8.27** | **5** | -6.11 | 20 | **-9.93** | **1** | **-10.02** | **1** |
| BDG 34017323 | -5.56 | 31 | -7.24 | 9 | -7.61 | 22 | -8.60 | 13 |
| BDH 33910157 | -5.63 | 29 | -7.26 | 7 | -8.35 | 6 | **-9.59** | **2** |
| BDH 33911533 | -6.95 | 12 | -6.05 | 22 | **-8.86** | **3** | **-9.16** | **4** |
| BDH 33920975 | -5.74 | 27 | -5.18 | 33 | -8.12 | 10 | -8.55 | 14 |
| BDH 33959077 | -7.27 | 10 | -6.77 | 14 | -6.50 | 32 | -6.88 | 34 |
| BDH 34000291 | **-8.37** | **2** | **-9.06** | **3** | -7.28 | 25 | -8.17 | 18 |
| BDH 34016510 | -5.72 | 28 | -6.08 | 21 | -7.89 | 17 | -7.55 | 31 |
| BDH 34016524 | -6.23 | 21 | -5.53 | 29 | -7.94 | 15 | -7.60 | 29 |
| BDH 34016667 | -4.80 | 36 | -5.15 | 34 | -8.08 | 12 | -7.98 | 22 |
| BDH 34016679 | -5.94 | 23 | -5.91 | 25 | -8.08 | 13 | -7.90 | 26 |
| BDH 34016680 | -4.81 | 35 | -4.80 | 36 | -7.46 | 23 | -7.92 | 25 |
| BDH 34017775 | -6.27 | 20 | -6.39 | 17 | -6.89 | 31 | -6.95 | 33 |
| BDH 34019036 | -7.96 | 6 | -6.75 | 15 | -8.09 | 11 | -8.76 | 10 |
| BDI 34000884 | -4.73 | 37 | -6.68 | 16 | -7.03 | 27 | -7.07 | 32 |
| BDI 34016890 | -6.42 | 18 | -6.37 | 18 | -8.02 | 14 | -7.92 | 24 |
| LAS 34154496 | -5.82 | 25 | -5.50 | 30 | -2.66 | 37 | -5.18 | 36 |
| LAS 51495152 | -5.26 | 34 | -6.23 | 19 | -3.67 | 36 | -4.24 | 37 |
| LAS 51497195 | -5.93 | 24 | -6.00 | 24 | -5.75 | 34 | -6.70 | 35 |
| BDF 34002917 | -6.64 | 15 | **-9.31** | **1** | **-9.29** | **2** | **-9.20** | **3** |
| BDG 33909837 | -7.90 | 8 | -4.37 | 37 | -7.28 | 26 | -8.14 | 19 |
| BDG 33920931 | -5.78 | 26 | -5.87 | 26 | -5.96 | 33 | -8.99 | 7 |
| BDH 33910183 | -5.57 | 30 | -4.96 | 35 | -6.95 | 30 | -8.27 | 17 |
| BDH 33910188 | **-8.35** | **3** | **-8.46** | **4** | -6.96 | 28 | -7.93 | 23 |
| BDH 33910196 | -7.10 | 11 | -7.26 | 8 | -8.26 | 8 | -8.78 | 9 |
| BDH 33911472 | **-8.30** | **4** | -7.23 | 10 | -7.41 | 24 | -7.98 | 21 |
| BDH 33911485 | **-8.92** | **1** | **-9.17** | **2** | -7.64 | 21 | -8.28 | 16 |
| BDH 33911495 | -6.10 | 22 | -6.81 | 13 | -7.66 | 20 | -9.02 | 6 |
| BDH 33911518 | -6.50 | 16 | -6.04 | 23 | -7.67 | 19 | -7.65 | 28 |
| BDH 33920767 | -5.50 | 32 | -5.82 | 27 | -7.91 | 16 | -8.85 | 8 |
| BDH 33920936 | -6.67 | 14 | -7.21 | 11 | -7.79 | 18 | -8.04 | 20 |
| BDH 33920962 | -7.30 | 9 | **-7.58** | **5** | -6.96 | 29 | -8.68 | 12 |
| BDH 34004126 | -7.93 | 7 | -7.48 | 6 | -5.55 | 35 | -8.72 | 11 |
| BDH 34012219 | -5.46 | 33 | -5.21 | 32 | **-8.66** | **4** | **-9.03** | **5** |
| BDH 34012595 | -6.85 | 13 | -5.33 | 31 | **-8.39** | **5** | -7.68 | 27 |
| BDH 34019992 | -6.33 | 19 | -5.65 | 28 | -8.31 | 7 | -7.55 | 30 |
